# Signaling dynamics of palmitate-induced ER stress responses mediated by ATF4 in HepG2 cells

**DOI:** 10.1186/1752-0509-7-9

**Published:** 2013-01-22

**Authors:** Hyunju Cho, Ming Wu, Linxia Zhang, Ryan Thompson, Aritro Nath, Christina Chan

**Affiliations:** 1 Department of Chemical Engineering and Materials Science, East Lansing, MI 48824, USA; 2 Department of Computer Science and Engineering, East Lansing, MI 48824, USA; 3 Cell and Molecular Biology Program, East Lansing, MI, 48824, USA; 4 Genetics program, East Lansing, MI, 48824, USA; 5 Department of Biochemistry and Molecular Biology, Michigan State University, East Lansing, MI, 48824, USA

**Keywords:** ATF4, Palmitate-induced ER stress, CREB1, Discrete dynamic model, Signal transduction

## Abstract

**Background:**

Palmitic acid, the most common saturated free fatty acid, has been implicated in ER (endoplasmic reticulum) stress-mediated apoptosis. This lipoapotosis is dependent, in part, on the upregulation of the activating transcription factor-4 (ATF4). To better understand the mechanisms by which palmitate upregulates the expression level of ATF4, we integrated literature information on palmitate-induced ER stress signaling into a discrete dynamic model. The model provides an *in silico* framework that enables simulations and predictions. The model predictions were confirmed through further experiments in human hepatocellular carcinoma (HepG2) cells and the results were used to update the model and our current understanding of the signaling induced by palmitate.

**Results:**

The three key things from the *in silico* simulation and experimental results are: 1) palmitate induces different signaling pathways (PKR (double-stranded RNA-activated protein kinase), PERK (PKR-like ER kinase), PKA (cyclic AMP (cAMP)-dependent protein kinase A) in a time dependent-manner, 2) both ATF4 and CREB1 (cAMP-responsive element-binding protein 1) interact with the *Atf4* promoter to contribute to a prolonged accumulation of ATF4, and 3) CREB1 is involved in ER-stress induced apoptosis upon palmitate treatment, by regulating ATF4 expression and possibly Ca^2+^ dependent-CaM (calmodulin) signaling pathway.

**Conclusion:**

The *in silico* model helped to delineate the essential signaling pathways in palmitate-mediated apoptosis.

## Background

Elevated serum free fatty acids (FFA) and hepatic lipid accumulation in non-adipose tissues can lead to cellular dysfunction or cell death, due in part to the diversion of unoxidized FFAs to nonoxidative pathways, resulting in lipoapoptosis [1]. Excess amounts of nonesterified FFAs that fail to convert to triglyceride in liver cells, enhance the risk for hepatocellular lipoapoptosis, a pathogenic feature observed in non-alcoholic steatohepatitis (NASH) [2]. The mechanisms involved in FFA-induced toxicity have remained unresolved, however recent studies suggest that hepatic lipoapoptosis arises predominantly from FFA-induced lipotoxic stress of intracellular organelles, in particular the endoplasmic reticulum (ER) and mitochondria [3].

The ER is one of the largest organelles in the cells, and perturbing ER homeostasis or inducing ER stress has profound effects on cell survival. Cellular perturbations, such as alterations in calcium storage in the ER lumen or an imbalance in the luminal-oxidizing environment will cause ER stress. This stress is sensed by the cells through three ER transmembrane proteins, inositol requiring enzyme (IRE) 1α, PKR-like ER kinase (PERK), and activating transcription factor (ATF) 6α [4]. They activate signaling processes to restore ER homeostasis, and are collectively termed the unfolded protein response (UPR). UPR signaling pathways coordinate cellular response by down-regulating protein translation, enhancing expression of ER chaperone proteins that promote protein refolding, and activating proteases involved in the degradation of misfolded proteins. When these corrective actions are insufficient to attenuate ER-stress, the UPR switches to a pro-apoptotic mode. However, the mechanisms regulating ER-stress-induced apoptosis have not been clearly defined.

Of the ER transmembrane proteins, PERK is involved in regulating both translation and transcription to return the folding demand and capacity to homeostasis. Like PKR (double-stranded RNA-activated protein kinase, also known as eIF2αk2), one of the eukaryotic translation inhibition factor 2α (eIF2α) kinases, PERK (eIF2ak3) also phosphorylates eIF2α at Ser 51, and phosphorylated eIF2α impedes global translation initiation to decrease the protein load in the ER [5]. However, activated eIF2α paradoxically favors an increase in the translation of the activating transcription factor-4 (ATF4), a member of the activating transcription factor/the cAMP-responsive element-binding protein (ATF/CREB) family of basic zipper-containing proteins [6]. ATF4 binds to cAMP response element (CRE; TGACGT(C/A)(G/A)) as a homodimer to induce transcription of many pro-survival genes that are involved in amino-acid metabolism, redox reactions, stress response and protein secretion [7,8]. Under sustained ER-stress, ATF4 activates CHOP (C/EBP-homologous protein; also known as GADD153) transcription by binding to the CCAAT/enhancer binding protein (C/EBP)/ATF response element (CARE) sequences, likely as heterodimers with members of the C/EBP family transcription factor. CHOP in turn inhibits the expression of anti-apoptotic Bcl-2 (B cell lymphoma 2) protein [9,10]. Concomitantly, CHOP forms a heteromeric complex with the phosphorylated c-Jun to bind the PUMA (p53-upregulated modulator of apoptosis) promoter and contributes to the upregulation of pro-apoptotic proteins [11].

Saturated long chain-FFAs, especially palmitic acid, have been implicated in ER stress-induced apoptosis in liver cells [12,13]. Since both transcriptional regulation and translational control of ATF4 plays a central role on ER stress-induced apoptosis, recent studies have focused on the regulation of the ATF4 gene mediated by palmitate. In H4IIE liver cells, a rat liver hepatoma cell line, palmitate has been shown to induce ER stress, as evident by increased mRNA levels of ATF4 [12-14]. Similarly human hepatocarcinoma cell lines, such as HepG2 cells, respond also to palmitate by elevating ATF4 mRNA and inducing ER stress [15]. However, with the highly dynamic nature of UPR signaling, it would be judicious to integrate signaling pathways which are involved in the regulation of ATF4 and further elucidate the dynamics of the main signaling axis for ER stress-induced apoptosis, PERK/eIF2α/ATF4. The complexity of the signaling pathways involved makes it difficult to experimentally test all possible interactions to determine which pathways in the signaling network are functional for a given condition. Therefore, we applied a discrete dynamic network model to integrate potential interactions and components of ER signaling and its feedbacks in HepG2 cells to gain a better understanding of how palmitate induces ER stress in liver cells. The predictions are confirmed with further experiments.

## Results

### The conventional signaling pathway (PERK/eIF2α/ATF4) cannot explain the prolonged ATF4-dependent ER stress induced by palmitate in HepG2 cells

**Figure 1:**
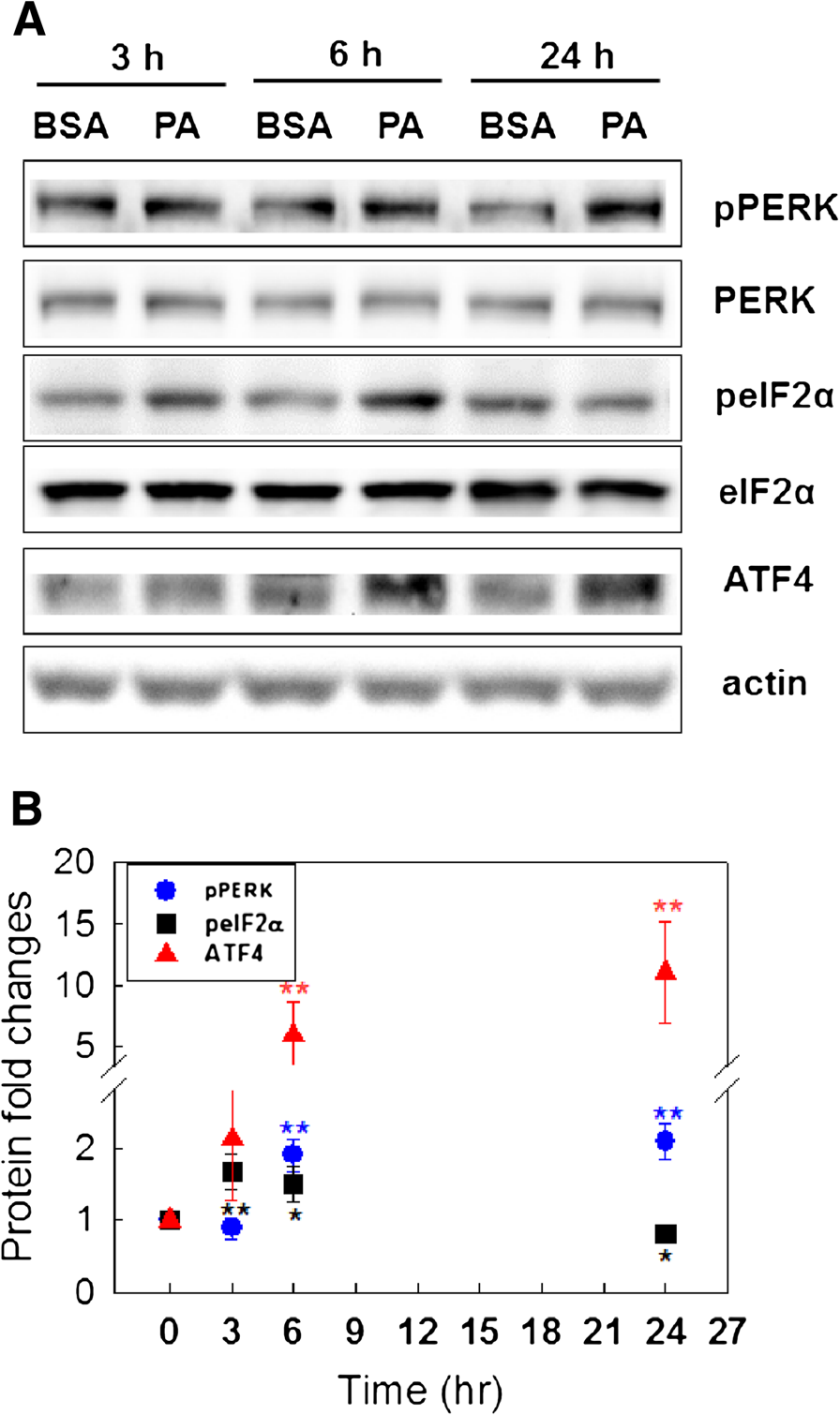
**Effects of palmitate on the PERK- eIF2α-ATF4 pathway. **(**A**) Western blotting analysis. HepG2 cells were treated with 2% BSA (as a negative control) and 700 μM Palmitate (PA). After 3, 6, 24 hrs, the cell extracts were collected and subjected to immunoblot analysis for total PERK (both phosphorylated and non-phosphorylated proteins), p-PERK (phosphorylated proteins), eIF2α, p-eIF2α, and ATF4. Actin served as a loading control. (**B**) Quantification. From Figure 1A, the phosphorylation levels of PERK and eIF2α were quantified and normalized to the total protein levels of PERK and eIF2α, respectively. The protein expression levels of ATF4 were also quantified. The protein fold changes were calculated at each time point using the following equation: (Protein expression level)_palmitate_/(protein expression level)_BSA_. Data represent the mean and standard deviation of three independent experiments: *p < 0.05 and **p < 0.01 vs. the control at each time point.

To evaluate the dynamics of the conventional ER signaling pathway, we measured the protein expression levels of PERK, eIF2α, and ATF4 at 3, 6, and 24 hrs upon palmitate treatment. As shown in Figure 1A, the phosphorylated protein levels of PERK at Ser 713 and eIF2α at Ser 51 were signficantly increased by palmitate treatment at 6, 24 hr and 3, 6 hr, respectively, while their total protein levels remained constant. Palmitate transiently increased the phosphorylation levels of eIF2α but induced a sustained increase in the protein expression level of ATF4. We note several discrepancies from the conventional signaling pathway. First, although eIF2α is activated transiently by palmitate, ATF4 continued to be strongly induced by palmitate, i.e. remained stably upregulated through 24 hrs (Figure 1B). In support of our results, a recent study also observed similar trends in a time-course analysis of eIF2α phosphorylation and ATF4 protein expression in HepG2 cells treated with thapsigargine, an ER calcium-ATPase pump inhibitor that induces calcium release from the ER [16]. The eIF2α phosphorylation was transientlty activated by thapsigarine and reached a maxium at 30 min, while ATF4 induction continued to rise for up to 8 hrs. However, it remains unclear why ATF4 remained stably upregulated even though the level of eIF2α phosphorylation was only transiently activated.

**Figure 2:**
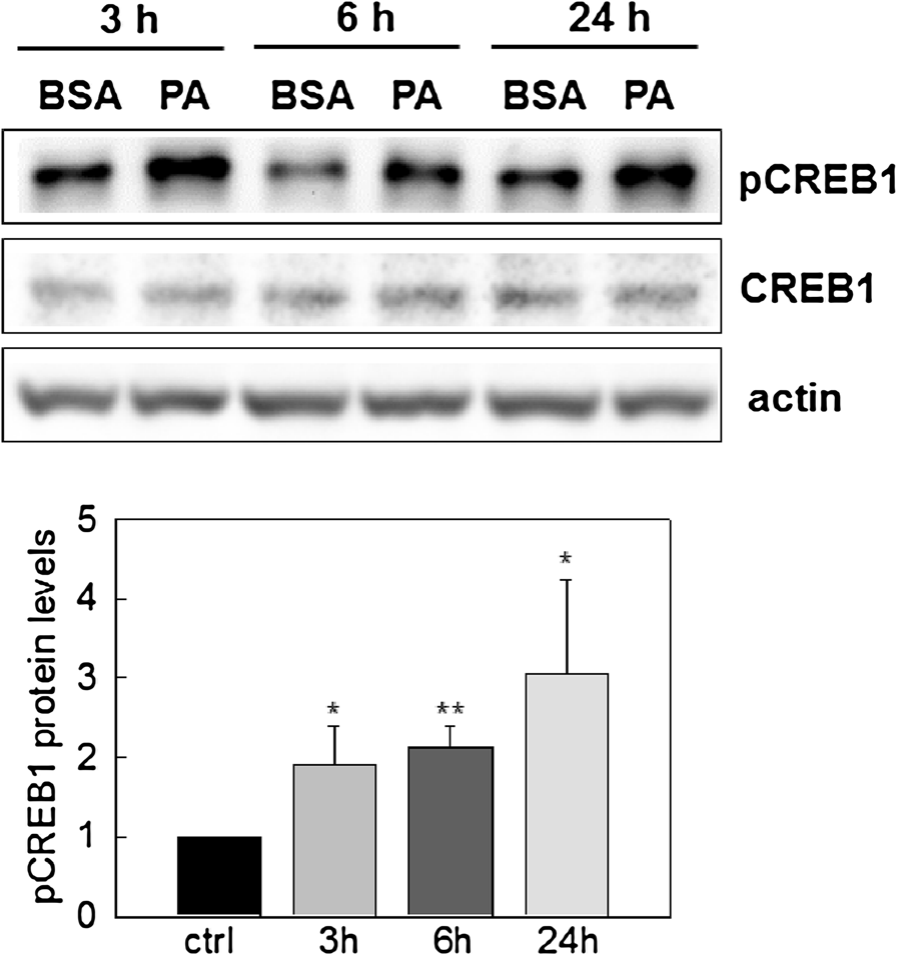
**Effects of palmitate on CREB1 phosphorylation. **Western blotting analysis. HepG2 cells were treated with BSA (as a negative control) and 700 μM Palmitate (PA). After 3, 6, 24 hrs, the cell extracts were collected and subjected to immunoblot analysis for total CREB1 and p-CREB1. Actin served as a loading control. The level of p-CREB1 was quantified by normalizing to the levels of total CREB and expressed as the average of three samples ± SD from three independent experiments: *p < 0.05 and **p < 0.01 vs. the control at each time point.

In addition, our group recently found that cAMP promoted apoptosis in palmitate-treated HepG2 cells, suggesting that cAMP-CREB1 signaling may be involved in palmitate-mediated ER stress [17]. Furthermore, it has been shown that the phosphorylation of CREB1 can be up-regulated by thapsigarine (ER stress inducer) in human glioma cells [18]. Since ATF4 (also known as CREB2) belongs to a family of ATF/CREB proteins, it raises a possibility that crosstalk exists between these two proteins during palmitate-induced ER stress. Therefore, we assessed whether the phosphorylation of CREB1 in HepG2 cells is up-regulated upon palmitate treatment. As shown in Figure 2, the phosphorylation of CREB1 was significantly induced by palmitate at all time points (3, 6, 24 hr).

**Figure 3:**
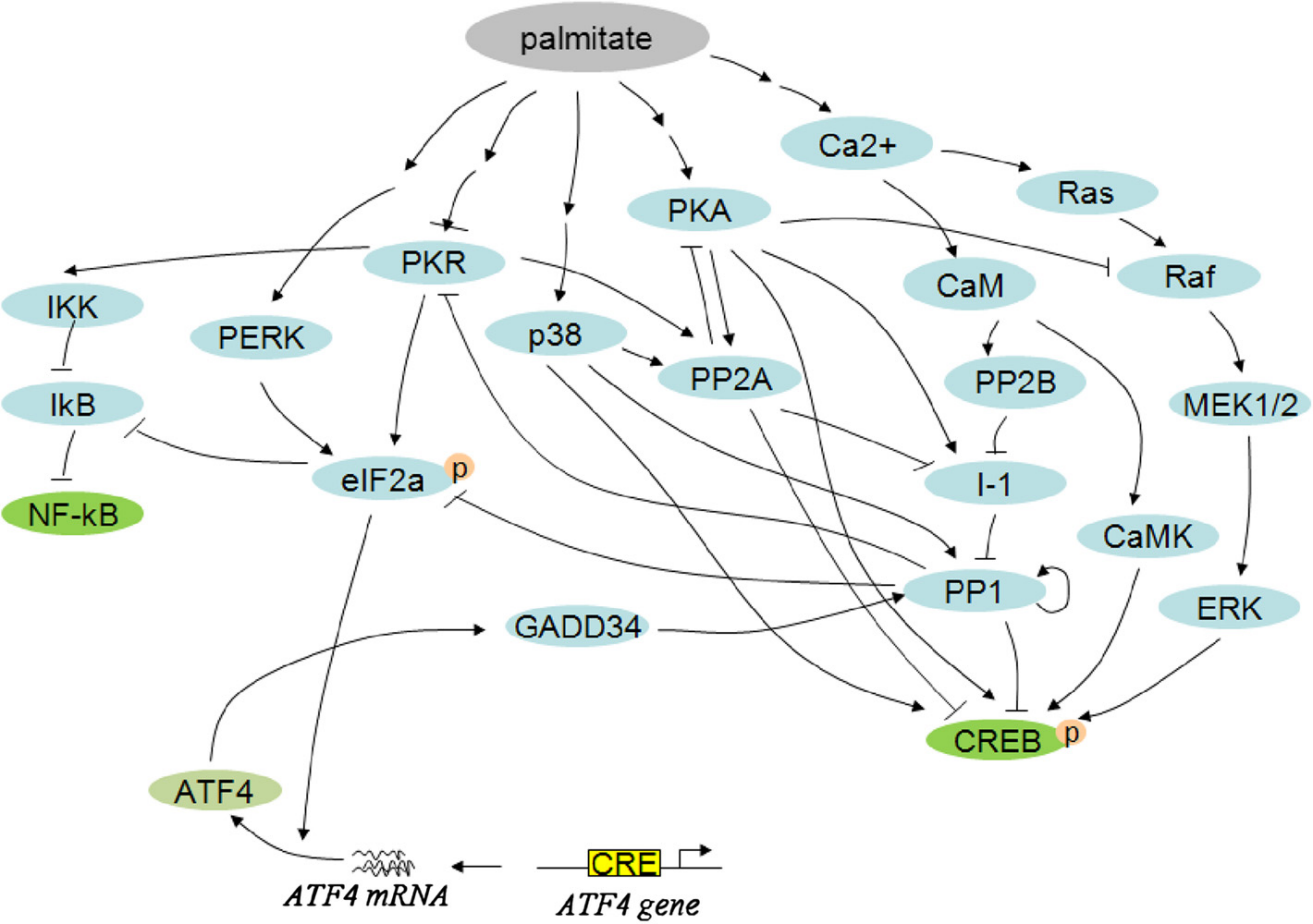
**Signaling network of ATF4-dependent ER stress mediated by palmitate. **Nodes are genes (proteins) possibly involved in the palmitate-induced signaling processes. Each arc represents a regulatory interaction (either activation or inhibition).

To resolve these discrepancies from the conventional pathway and incorporate our findings with the current knowledge, we collected and integrated information from the literature on palmitate-induced ER stress signaling, with an emphasis on how ATF4 could be activated by palmitate. We constructed a network of palmitate-induced signaling processes by collating the literature information (Figure 3). We formularized the signaling network into a discrete dynamic model. Our discrete dynamic modeling approach was previously proposed and successfully applied to dissect the insulin signaling pathways in the same human liver cell system [19]. We applied this modeling and simulation method to the palmitate-treated cells (see Methods), and integrated current knowledge to generate a signaling network, which was probed *in silico* to provide insight into the regulatory mechanisms involved.

### PKR pathway is essential for eIF2α phosphorylation in palmitate

**Figure 4:**
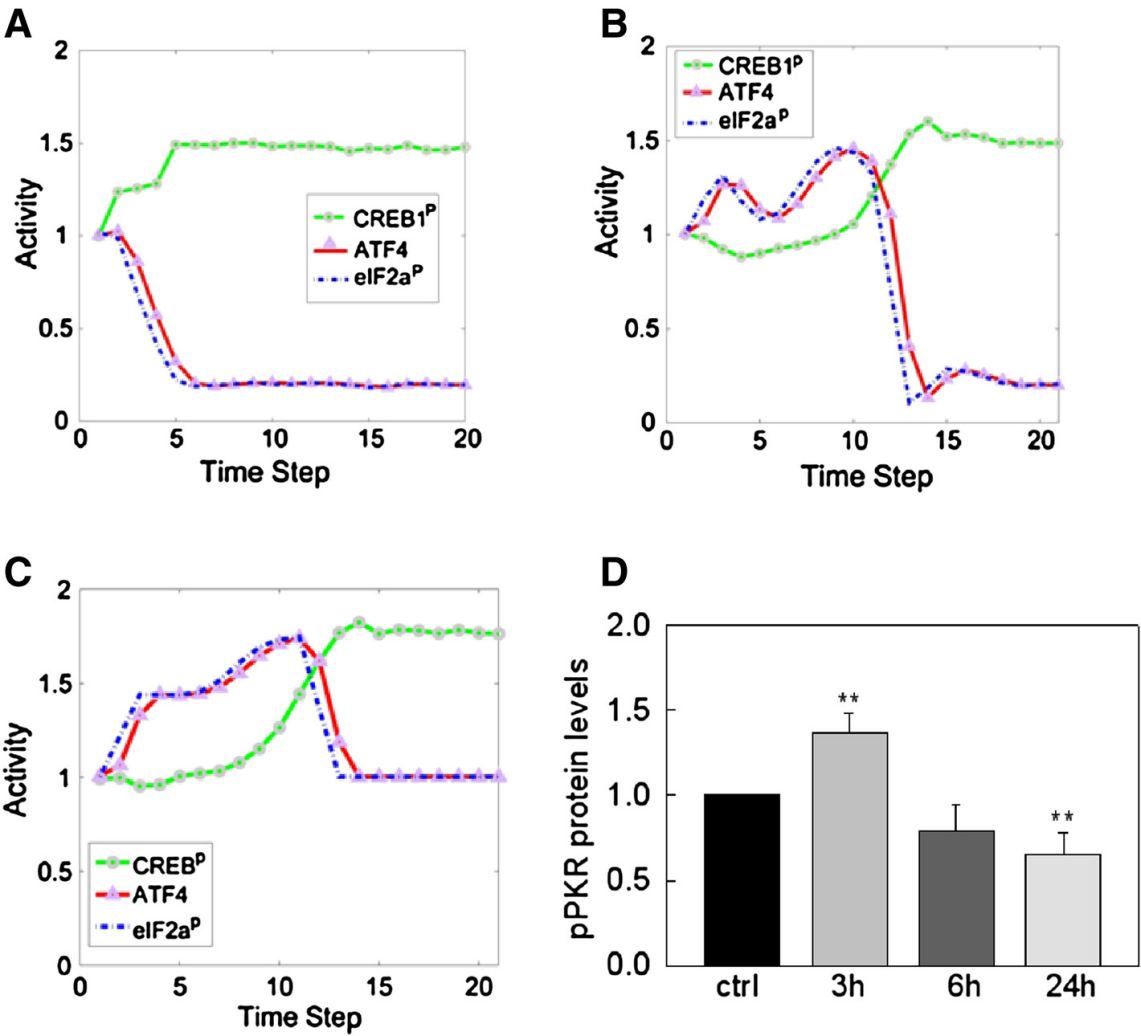
***In silico *simulation of the palmitate-induced signaling processes and effects of palmitate on PKR phosphorylation. **(**A**) Simulation model. The simulation is based on the systems model that includes the potential interactions and components from the literature, and we further incorporate new observations obtained from experiments to refine the model. The downstream signals emphasized are the level of eIF2α phosphorylation, CREB1 and ATF4 activity. Simulation results based on the network model in Figure 3 is an integration of the current literature knowledge. (**B**) The difference in the response time of PKR and PERK is added into the model. (**C**) The knowledge that PP1 activity is unchanged upon palmitate treatment is added into the model. (**D**) Western blotting analysis. The phosphorylated PKR level was measured using western blotting analysis after 3, 6, 24 hr of palmitate treatment of HepG2 cells. Actin served as a loading control. The level of p-PKR was quantified by normalizing to the levels of total PKR and expressed as the average of three samples ± SD from three independent experiments: **p < 0.01 vs. the control at each time point.

Our simulation results suggest plausible dynamic profiles of the network upon palmitate-stimulation (Figures 4A, 4B, 4C). The simulations are based on current knowledge of the regulatory interactions between the components in the network. We initially assume that the activation steps (mostly phosphorylation/de-phosphorylation) of the different components are at similar time scales. As shown in Figure 4A, CREB1 phosphorylation level was increased by palmitate over the simulation time, which matched the results obtained by western blotting analysis shown in Figure 2. However, the simulation results show that eIF2α and ATF4 were not activated by palmitate treatment (Figure 4A), which is inconsistent with the experimental results of Figure 1. The *in silico* results suggest an inconsistency with the current knowledge of the palmitate-induced signaling processes mediated by eIF2α and ATF4.

Since PKR, another eIF2α kinase, has been shown to be involved in ATF4-dependent ER stress in human embryonic kidney cells [20], we questioned whether PKR is involved in eIF2α phosphorylalion to regulate the pamitate-mediated ATF4 protein expression in HepG2 cells. The experimental results suggest that PKR shows a prompt response at 3 h (Figure 4D), while PERK takes longer to be activated (6 h) (Figure 1). In addition, since PKA (cyclic AMP (cAMP)-dependent protein kinase A) regulates CREB1 phosphorylation both directly and indirectly (through PP2A (nuclear protein phosphatase 2A)) [21,22], we measured the protein expression level of PKA upon palmitate treatment. The catalytic subunits of PKA, which plays an important role for PKA activation, were significantly upregulated at 24 hr (Additional file 1: Figure S1). Thus we concluded that palmitate initiates signaling at different times through PKR (3 hr), PERK (6 hr), and PKA (24 hr). We subsequently incorporated this new information into the discrete dynamic model, by imposing different delays in the palmitate-induced PERK and PKA activation. The resultant eIF2α profile (Figure 4B) matches the experimental observation (Figure 1) of a transient elevation in its phosphorylation level, which initiates the early ER stress response. The network model thus suggests an important role of the different response time of PKR vs. PERK in the regulation of the downstream effectors of palmitate. Thus we hypothize that the prompt response of PKR could initiate the signal that activates the eIF2α pathway.

### CREB1 may be involved in ATF4-dependent ER stress

Another significant discrepancy between the *in silico* simulation and the experimental observation lies in the prolonged activation of ATF4. The experimental measurements (Figure 1) show that ATF4 level is higher (than control cells without palmitate treatment) at both 6 h and 24 h. Such a prolonged activation cannot be explained by the model simulation where the ATF4 level is reduced to lower than control at 24 h, although the initial upregualtion of ATF4 in response to the upstream eIF2α is captured by the model. The discrepancy suggests the downsteam feedback regulation of ATF4 in the current model is incorrect in our liver cell system. The downstream feedback regulation in the model is mediated by phosphoprotein phosphatase 1 (PP1), which is known as a major regulator of ATF4 [23]. We measured the level of phosphorylated PP1 at different times upon PA treatment and found that, in contrast to current knowledge of the ATF4 pathway, there is no significant change on the activity of PP1 (Additional file 1: Figure S2) in our system that could affect the ATF4 level. The lack of involvement of PP1 explains in part the discrepancy between the current knowledge and our experimental observation of ATF4 activation. Indeed, when we updated our model with this new information of a constant PP1 level (i.e., to remove its impact on other components), the ATF4 profile is no longer “inhibited” in the simulation (Figure 4C). Nevertheless, the simulations are still unable to capture the prolonged activation of ATF4, because there is no other regulators that connect to ATF4 in the model to support its sustained activation after 6 h. This suggests that the current knowledge of the signaling process is incomplete and there should be other (currently unknown) regulatory relationship(s) in the network that could lead to the accumulation of ATF4 and finally lipoapoptosis.

**Figure 5:**
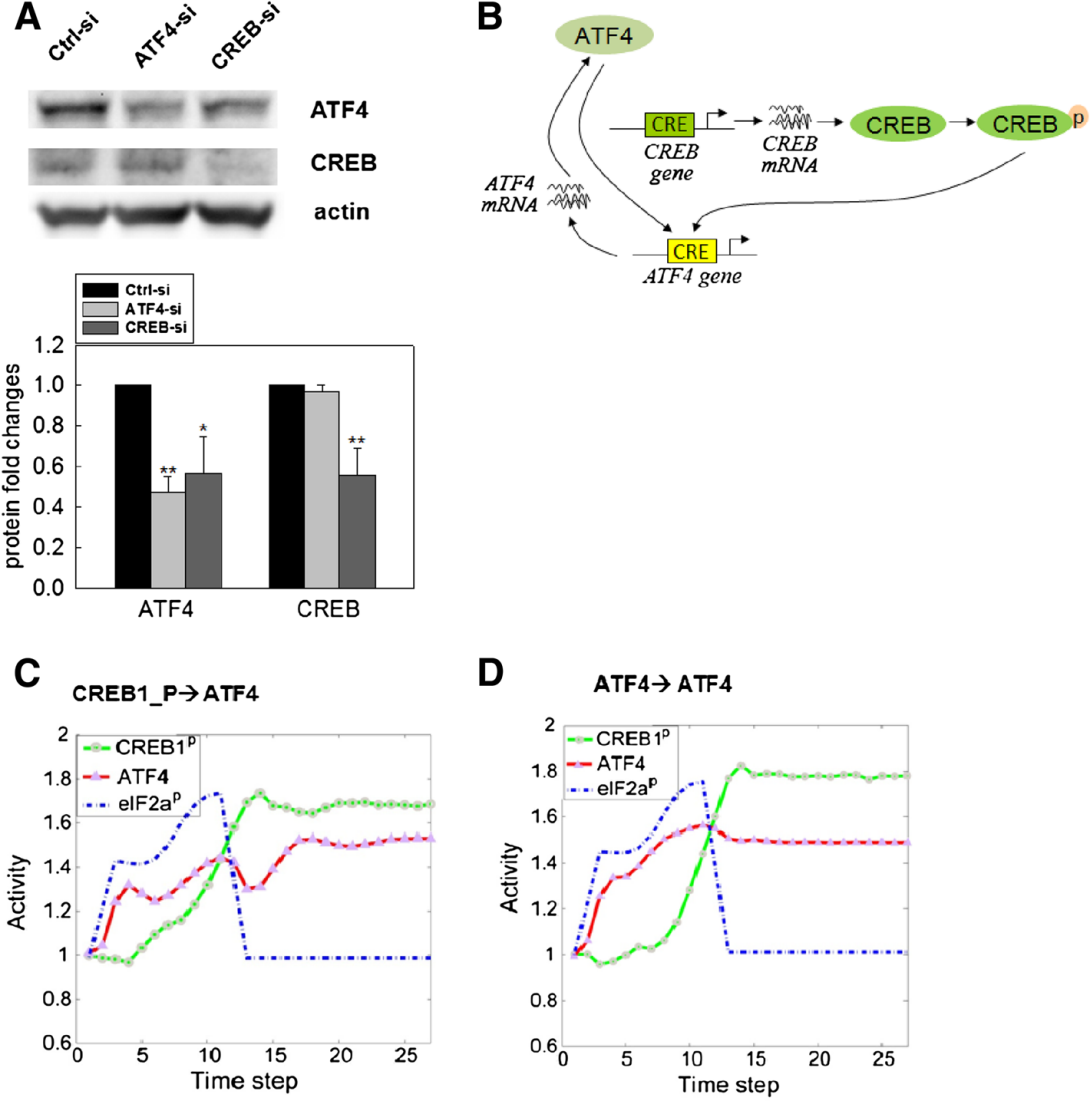
**ATF4-CREB1 interactions. **(**A**) Western blotting analysis. HepG2 cells were grown and transfected with ATF4 and CREB1 siRNA or negative control siRNA for 24 h as described in Methods. After another 48 hr of culture in regular medium, the cell extracts were collected and analyzed by western blot. Protein expression levels were quantified and displayed as fold-changes as compared to the loading control in the bar graph. Data are presented as means ± SD from three independent experiments: *p < 0.05 and **p < 0.01 vs. the control. (**B**) Potential model of ATF4-CREB1 interactions. (**C**) and (**D**) The simulation is based on the modified model that incorporates the results of Figures 4 and 5. The simulation results shows the predicted dynamic profile with (**C**) active CREB1 protein binding to the ATF4 promoter to induce ATF4 transcription, and (**D**) ATF4 protein binding to its own promoter to induce transcription.

CREB1 is of the same family as ATF4 and the phosphorylation of CREB1 was significantly enhanced upon palmitate treatment (Figure 2). Multiple CRE binding sites (TGACG or CGTCA) are identified on the ATF4 and CREB1 genes. Both CREB1 and ATF4 proteins could bind the putative CRE binding sites to enhance their gene expressions. Thus, we tested whether silencing either gene affects the protein expression level of ATF4 and CREB1. As shown in Figure 5A, CREB1 silencing significantly reduced the protein expression of ATF4, while ATF4 silencing did not significantly affect the protein expression level of CREB1. From these results, we hypothesized that both ATF4 and CREB1 regulate the gene expression of ATF4. This potential crosstalk (Figure 5B) between ATF4 and CREB1 pathway was integrated into our system model (Figure 3), and the simulation results (Figure 5C and 5D) predict a prolonged activation of ATF4 level, with either ATF4 binding to its own promoter, or CREB1 binding to the *Atf4* promoter to induce ATF4 gene expression.

### ATF4 protein binds to the CRE site on the Atf4 promoter *in vitro*

**Figure 6:**
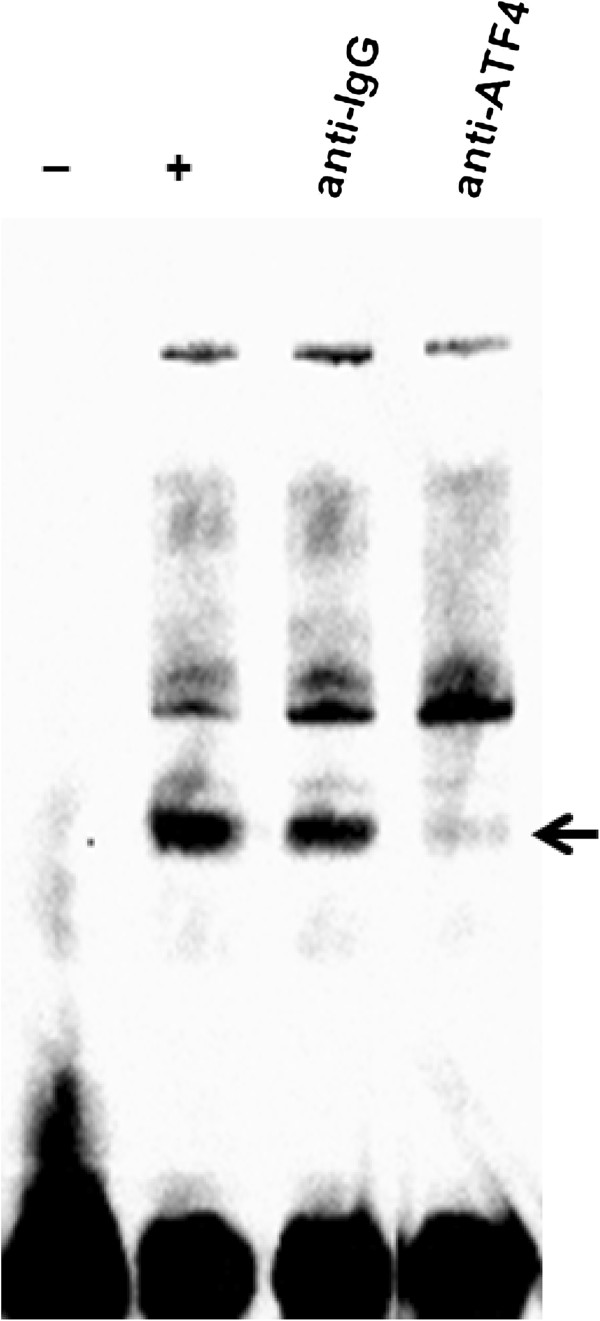
**EMSA of the *Atf4 *promoter region using palmitate-treated cell extracts. **Prior to nuclear extract isolation, HepG2 cells were incubated for 24 h in 0.7 mM palmitate conjugated 2% BSA. EMSA analysis to monitor binding to the CRE site (CGTCA; −164 to −160) was performed as described in Methods section. The first lane marked ‘−’ contains the probe alone and the second lane marked ‘+’ contains the probe and the nuclear extract. The remaining lanes contain antibodies in addition to the probe and the nuclear extract. Arrow represents a complex of ATF4-the DNA probe.

Since ChIP-chip data for pCREB1 are available, we determined whether the CREB1 protein interacts with the *Atf4* promoter. The hepatocyte-specific ChIP-chip data for pCREB1 binding on *Creb1* and *Atf4* promoters were obtained from the CREB1 target gene database [24]. A cut-off value of binding ratio ≥ 2 and confidence level p-value ≤ 0.001 was used to determine pCREB positive promoters (see ref [24] for details). ChIP-chip data showed that both ATF4 (binding ratio = 3.1, p-value = 4.1e-06) and CREB1 (binding ratio = 2.1, p-value = 8.3e-04) are positive for pCREB1 in hepatocytes, suggesting that the CREB1 protein directly binds on both the *Creb1* and *Atf4* promoters. In addition, to experimentally examine whether the ATF4 protein interacts with the *Atf4* promoter, we designed an ATF4 probe (-175 to -147) which contains one CRE site (CGTCA; -164 to -160) and performed an EMSA assay. As shown in Figure 6, the ATF4 probe formed a DNA-protein complex with the nuclear extract from the palmitate-treated HepG2 cells (lane 2; marked as an arrow). The IgG antibody does not cause any significant change of the DNA-protein complex (lane 3), while the ATF4 antibody eliminated the complex (lane 4). Since the disappearance of a band by the addition of a specific antibody, but without a supershift, provides evidence of DNA-protein complex [23,25], our results suggest that the ATF4 protein directly binds to the CRE site on the *Atf4* promoter. Thus, the ChiP-chip data and our EMSA data confirmed our potential models in Figure 5C and 5D, supporting that both ATF4 and CREB1 proteins could regulate the ATF4 gene expression.

### *In-silico* perturbation study suggests the essential signaling pathways for ATF4 and CREB1 activation mediated by palmitate

To identify the essential signaling pathways of ATF4 and CREB1 activated by palmitate, we simulated a model that incorporated our experimental findings and then evaluated the dynamics of eIF2α phosphorylation, ATF4 expression, and CREB1 phosphorylation, by systematically deleting specific pathways. We addressed whether PKA, p38, Ca^2+^ activation regulated by palmitate are essential for CREB1 phosphorylation. The *in silico* knock-out results (Additional file 1: Figure S3) showed that PKA-CREB1 and p38-CREB1 interactions did not alter the dynamics of the model. However, the deletion of Ca^2+^ signaling dramatically reduced CREB1 phosphorylation (marked by an arrow in Additional file 1: Figure S3). The model further showed that the PKA-, p38-, Ras-deficient model was able to capture the dynamic response of CREB1 and ATF4, as long as the Ca^2+^ signaling was able to induce CREB1 phosphorylation in response to the palmitate treatment. It has been shown that Ca^2+^ dependent-calmodulin (CaM) plays an important role in the activation of the CREB1 pathway [26,27], and our simulation results suggest that the calcium signaling pathway should be an essential component in the signaling network in response to palmitate stimulation of the liver cells. In other words, calcium signaling is sufficient to activate CREB1 phosphorylation, without the other pathways, and to recapitulate the dynamic behavior observed experimentally.

**Figure 7:**
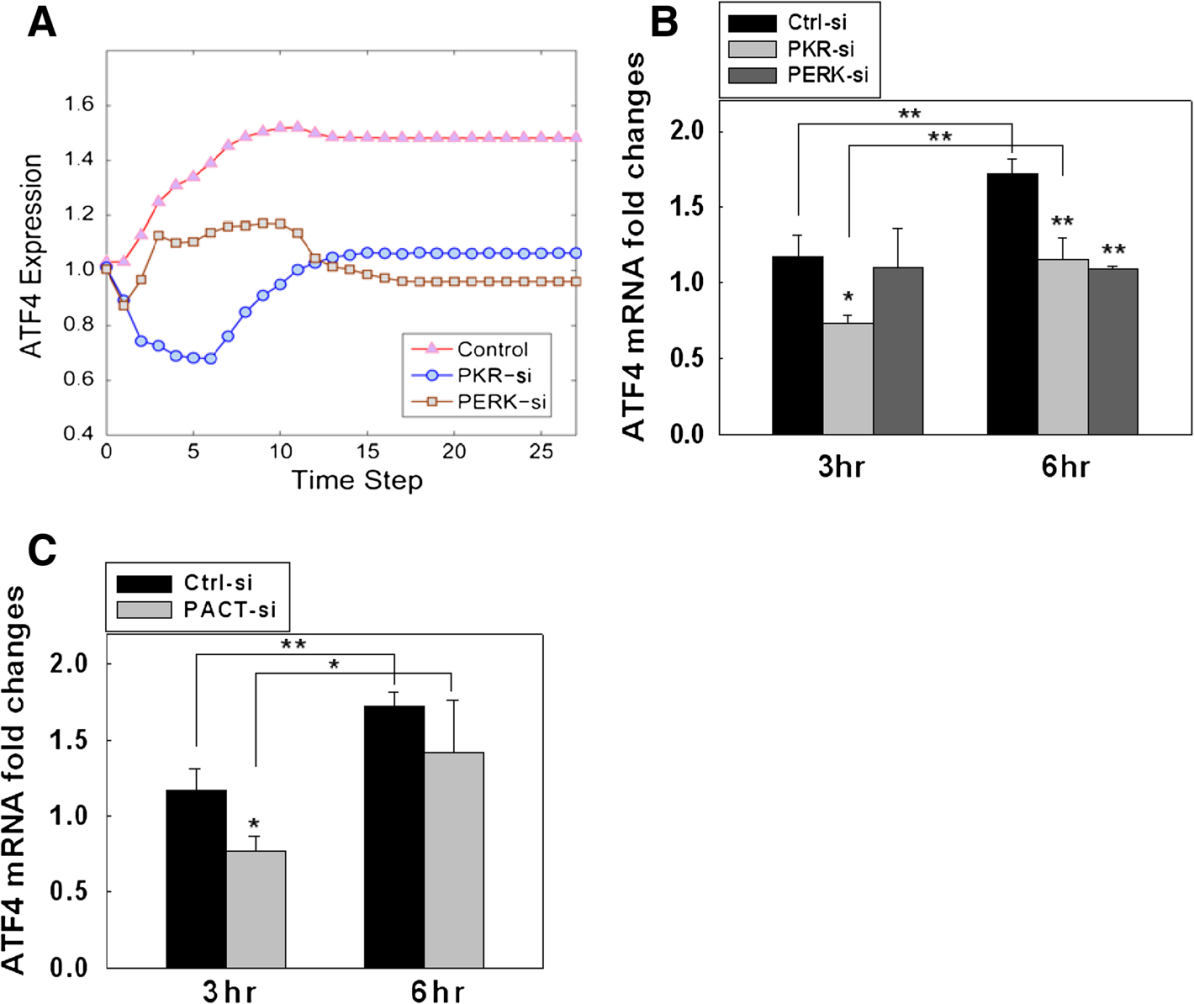
**Breakdown of the PKR-ATF4 or PERK-ATF4 signaling. **(**A**) *In silico *knock-out of PKR and PERK. The essential model includes PKR, PERK and Ca^2+ ^pathways as the downstream responders to palmitate stimulation. *In silico *simulation is performed on the essential model and compared with a model wherein either PKR or PERK is knocked-out. (**B**) and (**C**) Real-time quantitative RT-PCR analysis: (**B**) Knockdown of PKR and PERK gene expression and (**C**) knockdown of PACT gene expression. HepG2 cells were grown and transfected with siRNA of PKR, PERK, and PACT or negative control siRNA for 24 hr as described in the Methods. The cells were incubated for 3 and 6 hr in media containing BSA or BSA-complexed palmitate. The total mRNA were extracted and transcribed into cDNA. mRNA expression levels were quantified by real-time quantitative RT-PCR analysis and displayed as fold-changes as compared to the control samples treated with BSA in the bar graph. Data are presented as mean ± SD from three independent experiments: *p < 0.05 and **p < 0.01 vs the control at each time point. A line indicates comparison between the 2 bars connected by the line.

**Figure 8:**
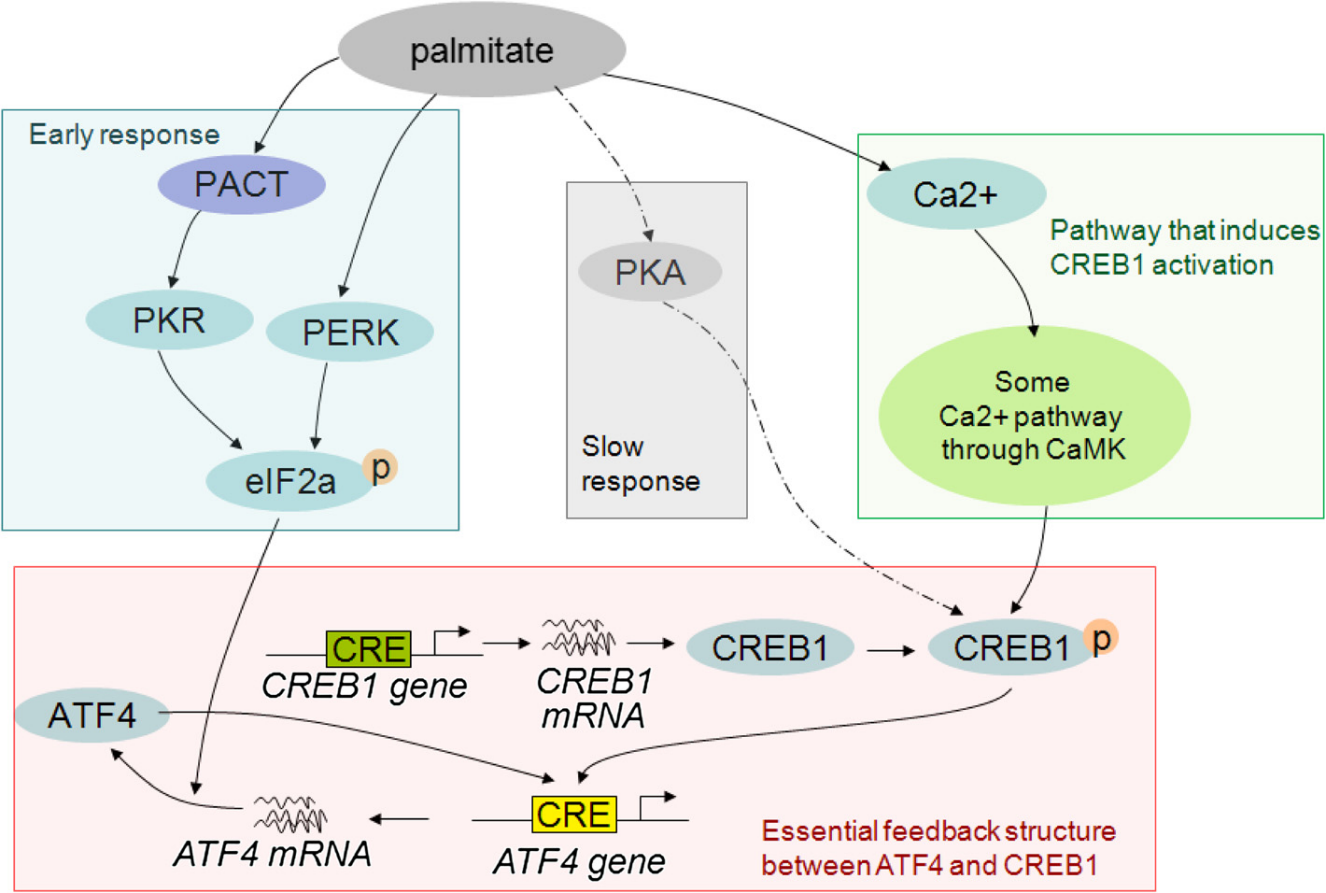
**Essential signaling network of palmitate-induced ATF4 expression. **The direct interactions between p38-CREB1 and Ras-CREB1 are deleted. The PP1 feedback to eIF2α is removed. Along with the essential feedback structure between ATF4 and CREB1, three main downstream signaling pathways are summarized: 1) Early response with PACT-PKR and PERK, 2) Slower response with PKA and 3) Ca^2+ ^signaling.

Since PKR and PERK are two main upstream signaling components of ATF4, we further evaluated whether knock-out of either PKR or PERK perturbs the signaling dynamics of ATF4. Simulation results (Figure 7A) suggest that the differences between PKR and PEKR perturbations (inhibitions) show up at an early time step (within 10 time steps, which correspond to ~6 hr in experimental time, according to the experiments in Figure 1 and the simulation results in Figures 4 and 5). The ATF4 level under PKR-silencing decreased much more significantly than the level under PERK-silencing in the earlier time point (e.g. at 5 time-steps, captured at ~3 hr in experimental time). This subtle difference comes from the underlying mechanism of the regulatory network where PKR and PERK plays different roles (i.e. palmitate initiates the PKR-eIF2α-ATF4 pathway through PACT-PKR activation at an earlier time then PERK), and is captured by the model. Our experimental results confirm this difference (Figure 7B and C), that PKR-knockdown cells showed significantly lower ATF4 gene expression level at the earlier time point (3 hr), while PERK-knockdown started to reduce the ATF4 gene expression level at a later time (6 hr). These results are consistent with the *in-silico* simulations (Figure 7A), suggesting that PKR is responsible for the early activation of ATF4. In addition, since ER stress in human embryonic kidney cells has been shown to involve the activation of PKR by PACT (PKR activating protein) [20], we experimentally tested whether PACT silencing blocks ATF4 mRNA expression levels. Treating the cells with palmitate for 3 hr resulted in a significant decrease in the ATF4 gene expression (Figure 7C), suggesting that the PACT-PKR pathway is essential for the early ATF4 gene expression, and possibly for an early ER stress response. Based on our computational analysis and experimental results, we identified the essential pathways involved in activating ATF4 and CREB1 in HepG2 liver cells upon palmitate treatment (Figure 8).

## Discussion

Increased long-chain saturated fatty acids, a characteristic feature of obesity and NASH [28], can induce ER stress, activate the UPR, and lead to cell death in hepatocytes [12,13]. Increasing number of studies implicate an involvement of palmitate in ER stress-induced apoptosis in liver cells [11-14,29,30], but the mechanism by which palmitate regulates the UPR signaling pathways is unclear. The present study sought to determine which signaling pathways are essential for the activation of ATF4, which plays a crucial role as a mediator of ER stress-induced apoptosis.

PKR, an eIF2α kinase, has recently emerged to be involved in ER stress-induced apoptosis by pharmacological ER inducers, thapsigargine and tunicamycin [20,31,32]. Lee *et al.* found that activated PKR phosphorylated the eIF2α in a PERK-independent manner when HEK293A cells were treated with thapsigargine [20]. They showed that PKR was responsible for approximately 40% and PERK for over 50% of the total phosphorylated eIF2a at 12 h upon thapsigargin treatment. However, our study suggests that the contributions of PKR and PERK on eIF2α phosphorylation change in a time dependent-manner upon palmitate treatment. At an earlier time (3 hr), PKR is entirely responsible for eIF2α phosphorylation (Figure 4D), with PERK adding to this contribution, to the eIF2α phosphorylation, at 6 hr (Figure 1). The relative contributions of PKR and PERK on eIF2α phosphorylation would also depend on the types of inducers and cells [20,33,34]. It would be worthy in future to investigate the kinetics of PKR and PERK activations to obtain more information on the roles of PKR and PERK-dependent ER stress.

Along with the positive regulation of PKR and PERK on eIF2α phosphorylation, a negative feedback loop could relieve the translational inhibition during ER stress, through GADD34-mediated PP1 activation [35-37]. ATF4 has been shown to bind specifically to a conserved ATF site on the GADD34 promoter in a stress-dependent manner. GADD34 in turn binds and activates the PP1 protein, which leads to eIF2α dephosphorylation and the resumption of general translation. In contrast to the conventional mechanism, we found that palmitate did not promote PP1 phosphorylation and the negative feedback mechanism was not activated in the palmitate-induced ER stress in HepG2 cells. Blockage of the negative feedback by palmitate may promote ATF4 accumulation in the cells while maintaining a normal level of eIF2α phosphorylation. In keeping with the concept of our study, a recent report suggested that salubrinal (well known as an eIF2α inhibitor) interrupts the feedback mechanism by inhibiting GADD34/PP1 complex activity, thereby potentiating palmitate-induced ER stress in pancreatic β-cells [38]. Similar to the synergistic roles of salubrinal and palmitate in β-cells, palmitate alone may be sufficient to induce ER stress-induced apoptosis in HepG2 cells [17,39].

ATF4 is known to form homo and heterodimers for DNA binding [40]. One study showed that ER stress induces a complex formation between endogenous ATF1/CREB1 and ATF4, subsequently binding to the ATF/CRE site of the *Grp78* promoter [41]. Along with the ChIP-chip data [24], one group recently suggested that CREB1 is able to bind the CRE site (CGTCA ; −921 to −917 ) on the *Atf4* promoter and a constitutively active PKA catalytic subunit dramatically activated *Atf4* promoter through PKA-CREB1 signaling [23]. Consistent with this study, our experimental and computational data showed that PKA activated by palmitate at a later time (24 hr) upregulates CREB phosphorylation, subsequently inducing the ATF4 expression. In addition, our EMSA data suggested that ATF4 protein binds its own *Atf4* promoter region containing the CRE site (CGTCA ; −164 to −160) and the positive-feedback stabilizes the ATF4 expression. Upon palmitate treatment, PKA or Ca^2+^-dependent CaM signaling pathways could enhance the interaction of CREB1 alone or the ATF4/CREB1 heterodimer with the *Atf4* promoter to prolong ATF4 accumulation. Although these remain open questions, our findings suggested that both ATF4 and CREB1 binding on the *Atf4* promoter plays an important role in prolonging ATF4 accumulation upon palmitate exposure.

In this study, our *in silico* model provides a framework to integrate regulatory information into a complex network and test the network for consistency by comparing the dynamic simulations with experimental measurements. Our simulations show inconsistencies between current knowledge of the network and our observation of palmitate-induced ER stress in the liver cells, which led us to explore the temporal response of the different pathways (e.g. PACT-PKR, PERK, PKA) to palmitate stimulation. This led to the identification of an essential feedback structure in the downstream ATF4/CREB1 regulation that differs from the conventional understanding of the mechanism. The study demonstrates an iterative learning process in which we begin by integrating the current knowledge to build a model to generate hypothesis, which is tested with experiments to obtain novel information. The new information is then incorporated into the network model to correct and update our understanding of the regulation. The updated model can be used to guide new experiments, thereby forming an iterative process that can systematically be applied to study biological processes. The discrete dynamic modeling approach developed in [16] for signaling network was expanded to account for transcriptional regulation by introducing delays into the regulation. New data and new information could be easily incorporated by adding delays (temporal information), altering gene activity states (perturbations with activator/inhibitor) or the network wiring (binding information), as demonstrated in this study of the regulatory network underlying palmitate-induced ER stress. This systems biology approach can be applied to other biological process to elucidate the molecular mechanism underlying the regulatory network by integrating experimental measurements and computational simulation.

## Conclusions

Integrating experiments and computational simulations helped to identify several notable findings, summarized in Figure 8. First, the dynamics of the signaling profiles show that palmitate initiates the PKR-eIF2α-ATF4 pathway through PACT-PKR activation at an earlier time and PERK later helps to maintain eIF2α phosphorylation. Second, ATF4 and CREB1 bind the *Atf4* promoter to contribute to a prolonged ATF4 accumulation and their feedbacks appear necessary for ER-stress induced apoptosis. Third, palmitate responds later to PKA activation and possibly along with the Ca^2+^ dependent-CaM signaling pathway to increase CREB1 phosphorylation. Thus this systems biology approach helped provide insight into the molecular mechanisms by which palmitate induces ER stress in liver cells.

## Methods

### The discrete dynamic modeling of biological network

We applied the approach of discrete dynamic modeling proposed in our previous study [19]. The model is constructed based on the topology and the regulatory relationships within a given network, with basic assumption that the network architecture defines the major dynamic characteristics of the system. We associate each component (protein) in the network with a discrete variable with three potential states (0: lower than control, 1: the control state, 2: higher than control), and simulate the system with transition rules (shift-up or shift-down) depending on the regulatory relationships between the components (activation or inhibition). More specifically, if an activator is in a state 2, (for example, a kinase is activated), the state of its target gene will be shifted upwards, from 0 to 1, or 1 to 2, depending on the target gene’s current state. In contrast, if the state of an inhibitor is higher than control, its target will be shifted downwards in the next updating event. The state of a component will decay (back to control state 1) if its regulators can no longer maintained their active state. Given this transition rules and a specific initial state of the system, we can compute a series of “state-changes” for each component along discrete time steps, where the current state of the system (i.e. the state of each component in the current time-step) depends on the both the previous states (i.e. states in the previous time-steps) as well as the transition process which imposes transition rules based on regulatory relationships within the network. Since the reaction rates may be different from cell to cell even for the same interaction, we apply asynchronous updating of the state, which is realized immediately, rather than renewing every variable simultaneously at each time-step. Thus, the relative rates of the different reactions can be specified by the ordering of the update, which implies that, although the response may be similar, the rate of response varies from cell to cell, or, between different runs. We perform 5,000 independent runs in each simulation to mimic a cell population that is measured by western blotting or RT-PCR experiments. We impose a distribution of initial states (centered at the control state 1) and a randomization of the ordering of the updates to represent cell-cell variation. The dynamic model was implemented by custom MATLAB code.

Two novel properties are introduced to the original modeling approach in [19] to extend the methodology to address different time-frames and to refine the model with posterior information (novel experimental observations).

1) Delayed processes. To deal with certain regulatory interactions that are expected to respond much slower than other signaling processes, such as transcription, translation, and the slower response of PERK upon palmitate treatment, we introduce a delay on these interactions such that the current state of the targeted gene depends on not the last state but an earlier previous state of its activator/inhibitor. Therefore, different from the original state transition: *S*_*i*_ *= f(S*_*i-1*_*)*, in which the current system state *S*_*i*_ depends on the system state of the last step *S*_*i-1*_, our new approach does not apply this Markov assumption, and instead *S*_*i*_ *= f(S*_*i-1,*_*S*_*i-2, …*_*S*_*1*_*)*, which could present a history-dependent dynamics. In practice, we implement the idea by simply introducing multiple intermediate (virtual) components in between the regulator and its targets to delay the signal transduction.

2) Encode posterior information into the model. Novel information, such as time-separation of the interactions, or the measurement of the activity of particular component in the network, could be incorporated to refine the model by adding constraints to the simulation. The constraints could be a delay of a certain interaction, or impose a constant value of activity on certain components (e.g. PP1) during the simulation.

The discrete dynamic model is based on the simple logic (activation/inhibition) in a regulatory network, which corresponds to our current (mostly qualitative) understanding in this biological system. We measure the average gene expression level in an “in silico” cell population that has varying initial states and order of reactions, to enhance the robustness of the simulation results. The Additional file 1: Figure S4 shows the increase in robustness of the model when a larger sample size is applied. There are variances in the different replicates of the simulation when the sample size is small, e.g. 50 runs (Additional file 1: Figure S4). These differences come from the randomness of the initiation (i.e. initial states) and the shuffling of the reactions, which are not related to the regulatory mechanism that is being modeled. By increasing the sampling size, the model becomes more robust. With 5000 runs, the different initial states or order of reaction have no effect on the averaged response curve, thus any differences that is observed between the different perturbations should reflect changes in the regulation imposed by the perturbation.

### Cell culture and reagents

HepG2/C3A human hepatocellular carcinoma cells were cultured in Dulbecco’s modified Eagle’s medium (DMEM) supplemented with 10% fetal bovine serum and 1% of penicillin–streptomycin (penicillin: 10,000 U/ml, streptomycin: 10,000 μg/ml; Invitrogen) in a humidified incubator at 37°C and 5% CO_2_. Sodium palmitate was purchased from Sigma. In all experiments, palmitate (0.7 mM) was complexed to 2% (w/v) fatty acid free BSA (US Biologicals) dissolved in regular medium. For palmitate treatment, HepG2 cells were seeded in the 6-well plate and cultured until 90% confluence.

### RNA interference and reverse transfection

SiRNAs targeting human PACT (5^′^-GAGAGAAUAUACUACAAUUTT-3^′^ and 5^′^-AAUUGUAGUAUAUUCUCUTT-3^′^), human PKR (5^′^-GGUGAAGGUAGAUCAAAGATT-3^′^ and 5^′^-UCUUUGAUCUACCUUCACCTT-3^′^), human PERK (5^′^-GUGACGAAAUGGAACAAGATT-3^′^ and 5^′^-UCUUGUUCCAUUUCGUCACTA-3^′^), human CREB1 (5^′^-CCGUAACUCUAGUACAGCUTT-3^′^ and 5^′^-AGCUGUACUAGAGUUACGGTG-3^′^) were purchased from Ambion. SiRNAs targeting human ATF4 (siGENOME SMARTpool #M-005125-005) was purchased from Dharmacon. The scrambled non-targeting siRNA (Ambion), used as a negative control, or the siRNA targeting proteins was diluted in Opti-MEM (Invitrogen) and then mixed with the transfection reagent, Lipofectamine RNAiMAX (Invitrogen). The mixture of siRNA and Lipofectamine RNAiMAX in Opti-MEM was then added into six well plates and incubated at room temperature for 20 min. 6 × 10^5^ HepG2 cells were suspended in antibiotic-free medium, seeded into 6-well plates, and cultured for 24 hr. After transfection, the cells were incubated in regular medium or palmitate-containing medium and then collected for further analysis.

### Real-time quantitative RT-PCR analysis

Total RNA was extracted from cells with the RNeasy mini kit (Qiagen). One microgram of total mRNA was reverse-transcribed using an iScript cDNA synthesis kit (Bio-RAD). The first-strand cDNA was used as a template. The primers used for quantitative RT-PCR analyses of human ATF4 (5^′^-TGGACTTCGAGCAAGAGATG-3^′^ and 5^′^-AGGAAGGAAGGCTGGAAGAG-3^′^) and human actin (5^′^-ACATCGCCCTGTGGATGACT-3^′^, and 5^′^-TCACTTGTGGCCCAGATAGG-3^′^) were synthesized by Eurofins MWG Operon. Amplifications of the cDNA templates were detected by SYBR Green Supermix (Bio-Rad) using RT-PCR Detection System (Bio-Rad) and the cycle threshold values were determined by the MyIQ software (Bio-Rad). Each sample was performed in triplicate and normalized to the actin expression levels.

### Western blot analysis

The HepG2 cells were washed twice with cold PBS and treated with CelLyticM cell lysis buffer (Sigma-Aldrich) or RIPA buffer (50 mM Tris (pH 8.0), 150 mM NaCl, 1% NP-40, 0.5% sodium deoxycholate, 0.1% SDS) supplemented with protease inhibitor cocktail (Sigma-Aldrich) for 10 min on ice. The cell lysate was clarified by centrifugation at 13000 rpm for 10 min, and the supernatant was collected. Total protein levels were quantified by Bradford assay (Bio-Rad). Thirty micrograms of total protein was loaded onto 9% SDS-PAGE gel, transferred to nitrocellulose membranes, and probed with antibodies for target proteins: PERK (Cell Signaling), p-PERK (BioLegend), eIF2a (Cell signaling), p-eIF2a (Cell signaling) ATF4 (Santa Cruz Biotechnology), CREB (Santa Cruz Biotechnology), p-CREB (Cell signaling), PKR (Novus Biologicals), and p-PKR (Novus Biologicals). The image was analyzed using the Molecular Imager ChemiDoc XRS System from Bio-Rad.

### Nuclear extract preparation and EMSA (electrophoretic mobility shift assay)

Nuclear extracts were prepared as described in the literature [42]. 0.7 mM palmitate-treated HepG2 cells (24 hr) were washed with ice-cold PBS, resuspended in Buffer (10 mM HEPES (pH 8.0), 1.5 mM MgCl_2_, 10 mM KCl, 1 mM DTT, 50 mM NaF, 1 mM orthovanadate, 1 complete protease inhibitor mini tablet/10 mL), and left to swell on ice for 15 min. Cells were then lysed by forcing them through a 25-gauge hypodermic needle 6-8 times. Lysate was centrifuged at 4000 g for 5 min to pellet the crude nuclear fraction. The nuclear pellet was then resuspended in Buffer (20 mM HEPES (pH 8.0), 420 mM NaCl, 1.5 mM MgCl_2_, 25% glycerol, 0.2 mM EDTA, 1 mM DTT, 50 mM NaF, 1 mM orthovanadate, 1 complete protease inhibitor mini tablet /10 mL) and incubated on ice for 30 min with gentle agitation. The nuclear extract was spun down at 16000 g for 15 min to pellet the nuclei. The supernatant was saved and used for EMSA.

A synthetic oligo representing −175 to −147 (ACTCCTTTTCTCGTCACAGCTACGCCCT) of the ATF4 promoter was used for EMSA. The probe was biotinylated with Biotin 3^′^-end DNA Labeling Kit from Thermo Scientific. EMSA method was modified from the previous study [43]. 5 μg of nuclear extract was incubated in EMSA buffer (10 mM HEPES (pH 7.9), 50 mM KCl, 1 mM MgCl_2_, 1 mM EDTA, 0.1% NP-40, 50 mM NaF, 1 mM Orthovanadate) with 2 μg of ATF4 antibody for 1 hr on ice. The biotinylated DNA probe was added to each mixture (with the DNA probe only as a negative control and the DNA probe with nuclear extract as positive control) and incubated for 30 min at room temperature. The reactions were loaded on a 4-20% TGX gel and separated by gel electrophoresis. The gel was then transferred on to a nylon membrane and UV crosslinked with a hand held crosslinker for 10 min at 254 nm. The probe was then visualized using the Chemiluminescencent Nucleic Acid Detection Module from Thermo Scientific.

## Abbreviations

ATF4: Activating transcription factor-4; Bcl-2: B cell lymphoma 2; CaM: Calmodulin; CARE: CCAAT/enhancer binding protein (C/EBP)/ATF response element; CHOP: C/EBP-homologous protein; CREB1: cAMP-responsive element-binding protein 1; EMSA: Electrophoretic mobility shift assay; eIF2α: Eukaryotic translation inhibition factor 2α; ER: Endoplasmic reticulum; FFA: Free fatty acid; IRE1: Inositol requiring enzyme 1; NASH: Non-alcoholic steatohepatitis; PACT: PKR activating protein; PERK: PKR-like ER kinase; PKA: cAMP-dependent protein kinase A; PKR: Double-stranded RNA-activated protein kinase; PUMA: p53-upregulated modulator of apoptosis; UPR: Unfolded protein response.

## Competing interests

The authors declare that they have no competing interests.

## Authors’ contributions

HC and LZ conducted all experiments except for EMSA. MW performed the computational simulations. RT carried out the EMSA experiments. AN participated in the analysis of ChIP-chip data. HC and MW drafted the manuscript. CC guided and directed the overall design and coordination of the study and edited the manuscript. All authors read and approved the final manuscript.

## Supplementary Material

Additional file 1 **Contains experimental and computational simulation results: Figure S1. **The protein expression levels of PKAc (PKA catalytic subunits) upon palmitate treatment, **Figure S2. **The protein expression levels of PP1 upon palmitate treatment, **Figure S3. **In silico knock-out of signaling pathways for CREB1 activation, **Figure S4. **Simulation of the discrete dynamic model with different amount of independent sampling size. (DOCX 763 kb) Click here for file
