# Supplementary material for: Signaling dynamics of palmitate-induced ER stress responses mediated by ATF4 in HepG2 cells
Source: BMC Syst Biol. 2013 Jan 22;7:9. doi: 10.1186/1752-0509-7-9 (PMC3557202; doi:10.1186/1752-0509-7-9)
Supplement: Additional file 1 — Contains experimental and computational simulation results: Figure S1. The protein expression levels of PKAc (PKA catalytic subunits) upon palmitate treatment, Figure S2. The protein expression levels of PP1 upon palmitate treatment, Figure S3. In silico knock-out of signaling pathways for CREB1 activation, Figure S4. Simulation of the discrete dynamic model with different amount of independent sampling size. (DOCX 763 kb) [file 1752-0509-7-9-S1.docx]

**Figure S1. The protein expression levels of PKAc (PKA catalytic subunits) upon palmitate treatment.**

HepG2 cells were treated with BSA (as a negative control), 400 µM and 700 µM Palmitate. After 3, 6, 24 hrs, the cell extracts were collected and subjected to immunoblot analysis for PKAc. GAPDH served as a loading control. The protein expression levels were quantified by normalizing to GAPDH levels and expressed as the average of three samples ± SD from three independent experiments: *p < 0.05.


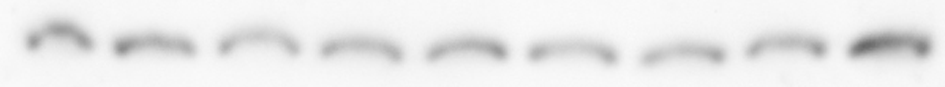

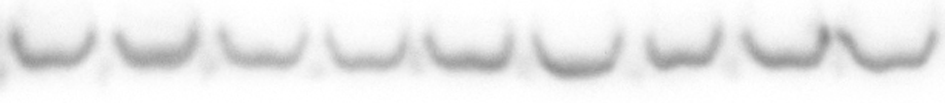


**3 hr**

**6 hr**

**24 hr**

**0 0.4 0.7**

**0 0.4 0.7**

**0 0.4 0.7 mM PA**

PKAc

GAPDH

**Figure S2. The protein expression levels of PP1 upon palmitate treatment.**

The PP1 levels were quantified as described in Figure S1.


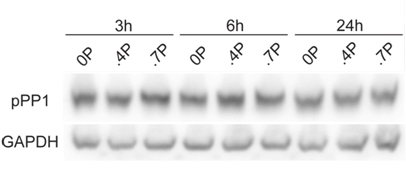


**Figure S3. *In silico* knock-out of signaling pathways for CREB1 activation.**

The full model includes the interactions in Figure 3 as well as ATF4 self-feedback and ATF4-CREB1 binding. *In silico* simulations show the dynamic profiles of eIF2α, ATF4 and CREB1 activity upon palmitate treatment. All simulations incorporated experimental evidences of different response time of PKR, PERK and PKA to palmitate and a constant level of PP1. To identify the essential pathways for CREB1 activation, we “knocked-out” the regulators in each pathway that lead towards CREB1 phosphorylation, including PKA, P38, Ras, and CaM, by removing them from the network model, and simulate the dynamic responses upon palmitate treatment to determine if there are significant changes in the dynamic profiles. The results show that these regulatory pathways could be redundant in the network model except when the Ca^2+^ dependent-CaM pathway is blocked, in which case the CREB1 phosphorylation is inhibited.

**
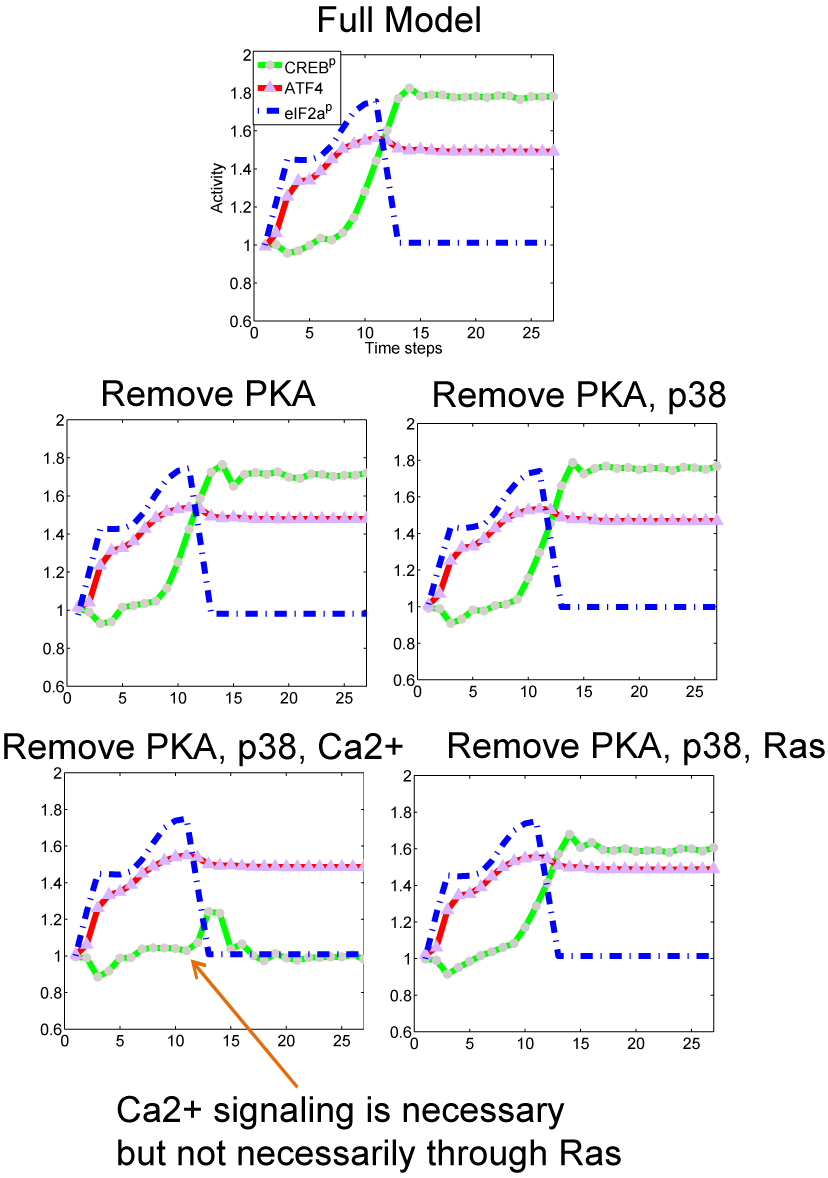
**

**Figure S4. Simulation of the discrete dynamic model with different sampling sizes.**

Simulations of CREB1 and ATF4 activity with 50 runs, 500 runs, and 5000 runs, and two replicates are presented for each simulation.

**
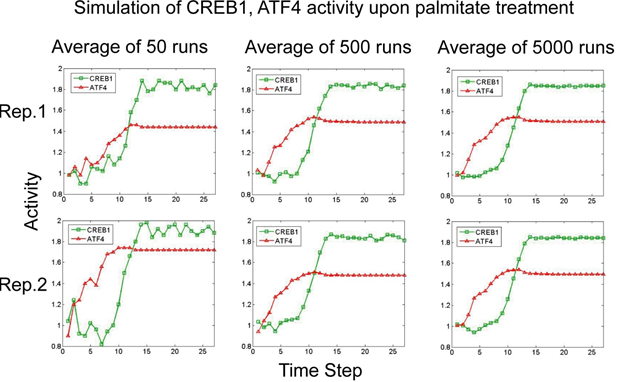
**
